# Transfusion-Transmitted Disorders 2023 with Special Attention to Bone Marrow Transplant Patients

**DOI:** 10.3390/pathogens12070901

**Published:** 2023-07-01

**Authors:** Miklós Udvardy, Árpád Illés, Lajos Gergely, László Imre Pinczés, Ferenc Magyari, Zsófia Simon

**Affiliations:** Division of Hematology, Department of Internal Medicine, Faculty of Medicine, University of Debrecen, 4032 Debrecen, Hungary

**Keywords:** transfusion, transmission, viral transmission, bacterial transmission, hematopoietic stem cell transplantation

## Abstract

Transfusion medicine is traditionally a strong/fundamental part of clinical practice, saving hundreds of millions of lives. However, blood-borne or transmitted infections are a well-known and feared possibility, a risk we relentlessly mitigate. Pathogens are continuously and rather quickly changing, so during the last decade, many, sometimes exotic, new pathogens and diseases were recorded and analyzed, and some of them were proved to be transmitted with transfusions. Blood or blood component transfusions are carried out after cautious preparative screening and inactivation maneuvers, but in some instances, newly recognized agents might escape from standard screening and inactivation procedures. Here, we try to focus on some of these proven or potentially pathogenic transfusion-transmitted agents, especially in immunocompromised patients or bone marrow transplantation settings. These pathogens are sometimes new challenges for preparative procedures, and there is a need for more recent, occasionally advanced, screening and inactivation methods to recognize and eliminate the threat a new or well-known pathogen can pose. Pathogen transmission is probably even more critical in hemophiliacs or bone marrow transplant recipients, who receive plasma-derived factor preparations or blood component transfusions regularly and in large quantities, sometimes in severely immunosuppressed conditions. Moreover, it may not be emphasized enough that transfusions and plasma-derived product administrations are essential to medical care. Therefore, blood-borne transmission needs continued alertness and efforts to attain optimal benefits with minimized hazards.

## 1. Introduction

Transfusion medicine and therapy with blood products are essential to clinical care and are indispensable tools that save millions of lives worldwide. On the other hand, they also carry the risk of blood-borne diseases and transfusion-transmitted infections, which themselves are significant, aside from the immunological risk the transfusion can mean [1,2].

This review, authored by a team of clinical hematologists, emphasizes the importance of coordinated and continuously updated clinical transfusiology. The team acknowledges the significance of various steps in preparative blood banking, such as donor interviews, pathogen detection, and implementation of inactivation measures, to ensure high standards of vigilance and safety. However, the evolving landscape of known and potential pathogens, influenced by travel and lifestyle changes, necessitates close cooperation among all stakeholders in clinical transfusiology. Additionally, advancements in preparative efforts, such as chilled platelets, pose significant challenges. The use of cooled products to reduce the risk of platelet transfusion transmission alters the primary hemostatic properties of the platelets. This review aims to provide a comprehensive summary of transmission data and emphasizes the need for vigilant monitoring of new information and its application in national clinical transfusiology. It underscores the importance of deepening the partnership between blood banking experts and clinicians, aiming for effective collaboration and optimal outcomes. In particular, patients undergoing bone marrow transplantation are at heightened risk and require a meticulous approach when considering transfusion and donor cell transmittable diseases.

## 2. Bacterial Transmission

Bacterial transmission is much more common than well-recognized and documented viral transmission [1,2,3]. The U.S. Food and Drug Administration (FDA) registered two to three fatal cases per year, recently [1,4].

Traditionally, packed red blood cell concentrates are not extremely dangerous from this aspect. The estimated number of blood-borne bacterial transmissions is around 1 per 38,000 transfusions. This seemingly marginal risk is achieved by careful donor screening, high-standard preparative procedures, and precautionary storage conditions (low temperatures of 4 ± 2 °C) [5]. Also, closed collection systems and disposable equipment have proven effective in preventing bacterial contamination of blood products.

The risk is much higher with platelet transfusions, a phenomenon especially important in oncohematology and bone marrow transplant patient care. The estimated transmission rate of bacterial infections is 1 per 2–5000 units of platelet transfusions with pooled, whole-blood-derived platelets and 1 per 15,000 units of platelet transfusions with single-donor apheresis preparations [1,6,7,8,9,10,11,12,13]. This substantial difference is primarily due to the storage of platelet products at room temperature (22 ± 2 °C). Documented cases involve four patients who developed bacterial sepsis following transfusion with single-donor apheresis platelet compounds containing *Acinetobacter calcoaceticus-baumannii* complex (ACBC) and *Staphylococcus saprophyticus* [12,14,15,16].

Recently, there have been attempts to utilize platelet cooling to mitigate the transmission of microbiological agents through platelet transfusions. However, despite some promising results, there are still unresolved issues that require further investigation. One concern is that chilled platelets have shown a propensity to clump more easily in laboratory settings, potentially affecting their function. Additionally, their aggregability appears to be heightened, although they tend to be cleared from circulation at a faster rate, possibly due to their enhanced binding to the vascular wall. Moreover, these cooled platelets exhibit increased activity in primary hemostasis as they bind more intensely to the endothelium. It is worth noting that the specific effects may vary depending on whether the platelets are derived from whole blood or obtained through apheresis. Consequently, these considerations highlight the importance of selecting the most appropriate platelet transfusion preparation method based on the clinical context, such as acute bleeding, prophylaxis, or the patient’s immunocompromised status, in the future [1,13,17,18,19].

*Treponema pallidum* can also be transmitted by transfusions; however, it does not cause manifest disease in the donor and seems to be extremely rare [3,20]. The transmission of *Treponema pallidum* through blood transfusion has become extremely rare due to the utilization of stored and refrigerated blood. However, *Treponema pallidum* infection continues to be a significant public health concern in many countries. Consequently, it is not uncommon to detect positive results for *Treopnema pallidum* screening tests among blood donors, which necessitates the implementation of preventive measures. In addition to their primary task, screening protocols for infections among blood donors are an effective approach to control the spread of diseases.

*Yersinia* transmission needs particular attention, because the symptomology is frequently non-characteristic, with moderate gastrointestinal discomfort. Moreover, *Yersinia enterocolitica* tolerates lower temperatures well and is therefore resistant to extended storage conditions [1,3,4,20].

There are significant geographical differences in the risk of bacterial transmissions worldwide [1,3,4,17,20]. It is essential to know that bacterial transfusion might induce acute reactions, very similar to transfusion reactions [13,21,22]. These different syndromes have practically identical symptoms, although they differ in terms of prevalence. The only way to reliably distinguish between the different syndromes is through confirmatory laboratory tests (Table 1).

## 3. Protozoal Infections

### 3.1. Malaria

Malaria is caused by five known species of *Plasmodium* that infect humans: *Plasmodium falciparum, Plasmodium vivax, Plasmodium malariae, Plasmodium ovale, and Plasmodium knowlesi*. Among these, *Plasmodium falciparum* is the most lethal, while *Plasmodium vivax* and *Plasmodium malariae* are also commonly detected in transfusion-transmitted malaria (TTM) [23,24].

Malaria parasites are naturally transmitted to humans through the bites of female *Anopheles* mosquitoes. Upon infection, *Plasmodium* invades and multiplies within human red blood cells, resulting in a potentially fatal acute febrile illness. The complex life cycle of the parasites involves a period of asymptomatic infection, allowing time for the host’s immune system to develop a protective response. TTM occurs when malaria parasites are transmitted directly through infected blood transfusions. This route of infection bypasses the initial asymptomatic phase observed in natural mosquito-transmitted malaria. TTM poses a higher risk of severe complications and can be fatal, particularly in non-immune individuals, such as young children and pregnant women in endemic areas or malaria-naïve adults in non-endemic regions [25].

The viability of *Plasmodium* parasites in stored blood is notable, as they can remain infectious for at least a week and potentially longer, depending on storage conditions. Even after 28 days of storage at 4 °C, microscopically detectable malaria parasites can persist, although their infectivity decreases after two weeks. The mean incubation period for TTM is generally longer than that of mosquito-transmitted malaria for all *Plasmodium* species [26]. Experimental evidence suggests that as few as 10 infected red blood cells can transmit the infection through TTM, highlighting the potential infectiousness of even a small inoculum [27]. Leukodepletion does not help in reducing malaria transmission rates [3,28]. Hemovigilance criteria, defined by the World Health Organization (WHO) and adapted to national guidelines, play a crucial role in monitoring and managing TTM risks in blood transfusion practices.

### 3.2. Babesiosis

Babesiosis, primarily caused by *Babesia microti*, is a tick-borne zoonotic disease transmitted through the bite of infected *Ixodes* species. However, it can also be transmitted through blood transfusion and solid organ transplantation when the donor is infected with *Babesia* [29]. Transfusion-transmitted cases of babesiosis are reported throughout the year, with the United States having the highest prevalence, primarily driven by *Babesia microti* infections in the Northeast and Upper Midwest regions. In certain western U.S. states, other *Babesia* species and related organisms are also implicated in the transmission of *Babesia* [30].

The majority of *B. microti* infections are asymptomatic and often go undiagnosed, while acute infection may induce hemolysis or thrombocytopenia, severe symptoms that can contribute to severe or even fatal cases. Individuals with asymptomatic infections can still have a period of parasitemia lasting from two to seven months, and in some cases, it can persist for over two years. Transmission of the infection and subsequent potentially fatal clinical illness can occur through the transfusion of blood products collected from asymptomatic donors [31]. Awareness of transfusion-transmitted babesiosis has increased in recent decades. The clinical presentation and severity of transfusion-transmitted babesiosis can vary, and babesiosis can lead to fatal consequences. An algorithm combining immunofluorescence assay (IFA) and polymerase chain reaction (PCR) has shown effectiveness in screening blood donations [32,33]. However, there is still an incomplete understanding of *Babesia* biology, and as the geographic range of *Babesia* and tick vectors expands, risk mitigation strategies may need to evolve.

### 3.3. Trypanosoma cruzi

*Trypanosoma cruzi* (*T. cruzi*) infection (Chagas disease) causes lymph node enlargement and irreversible organ damage, particularly affecting the heart and gastrointestinal tract. *T. cruzi* persists for a lifetime within host cells. However, it remains undiagnosed in most cases, with only 1% of patients receiving treatment in the acute or chronic phases. *T. cruzi* infection is endemic in some parts of Latin America. The transmission of *T. cruzi* through blood transfusion is a risk recognized by the Centers for Disease Control and Prevention (CDC), and screening tests have been approved by the U.S. Food and Drug Administration (FDA) [34]. Due to international migration, an increasing number of people infected with *T. cruzi* live worldwide, where seroprevalence studies have not been conducted yet. The WHO strongly recommends implementing preventive measures to avoid transmission in the European region [35]. Not all patients who receive infected blood components will become infected, due to reduced parasitemia or antibodies in the recipient [36]. A unique ELISA is needed to detect this agent [37]. *Trypanosoma brucei gambiense*, which is endemic in Africa, causes a disease known as sleeping sickness that exhibits similarities to the Chagas disease caused by *T. cruzii*. The transmission of sleeping sickness occurs primarily through the bite of the tsetse fly. While theoretically possible, cases of transmission through blood transfusion are rare and have rarely been documented.

## 4. Viral Transmission

### 4.1. HIV, Hepatitis B and C

Infections with HIV and hepatitis B and C viruses (HBV, HCV) can be transmitted via blood and cause severe acute or chronic liver infections. To ensure the safety of blood donations and protect recipients from virus transmissions, blood donations should be tested for viral genomes using nucleic acid amplification techniques (NATs) as well as for viral antigens and antibodies by serological testing. Pathogen inactivation seems to be a powerful tool with hepatitis B and C. Transfusion transmission of these pathogens is understood and thoroughly discussed in the literature [38,39,40,41,42,43]. Therefore, further evaluation is beyond the scope of this overview.

### 4.2. Hepatitis A and E

More recent data are available on hepatitis A and E viral transmissions [14,44,45,46,47]. Both viruses spread mainly via the fecal–oral route, but E virus infection develops from raw or cooked pork food exposure or even within a transfusion setting if the donor had hepatitis A or E viremia at donation. Hepatitis E viruses G1 and G2 are typical fecal–oral transmission agents, especially in developing countries. G3 and G4 strains of the hepatitis E virus can be considered as a zoonosis spread by foods of pork origin [46,47]. These viruses are resistant to two plasma component fractionation or solvent detergent inactivation. The hepatitis E virus issue seems to be more critical since health care personnel usually are less vigilant with this virus in general and its transmission by transfusion procedures. It became apparent during the last couple of years that E virus-induced hepatitis is pretty common in the U.K. and other European countries, and as a consequence, this virus can be transmitted with the transfusion or blood components outside of endemic areas [46,48,49]. Therefore, it is essential to screen the donors and, in the meanwhile, further identify screening methods for hepatitis virus safety. Hepatitis E virus serology is a relatively simple way to search antibody response; probably IgM positivity indicates acute infection and viremia, so this person cannot be a donor until this positivity ends. IgG positivity indicates previous infections. In this case, viral persistence (lymphocytes, hepatocytes) might sometimes be present. Therefore, direct E virus PCR would be desired to provide reliable data on transfusion safety. This PCR is still not easily and readily available, but proteinuria, chronic liver disease markers, splenomegaly, and blood count abnormalities (hemolysis, cytopenia) might also call attention to E virus persistence [44,45,46,48]. The success of the recent introduction of mandatory HEV NAT testing in 2020 have to be summarized in the upcoming years.

### 4.3. Zika

Zika, a flavivirus, is primarily transmitted by *Aedes* mosquitoes, particularly *Aedes aegypti*, which is also responsible for transmitting dengue (another flavivirus) and chikungunya (an alphavirus). Other modes of Zika virus transmission include intrauterine, perinatal, and sexual routes. The contribution of sexual transmission to the overall Zika virus risk is unknown. Zika virus was initially identified in Africa and has subsequently spread to Asia [50].

The risk of Zika virus transmission through the blood supply remains unclear, but evidence suggesting the potential for transfusion transmission emerged during the French Polynesian outbreak when 2.8 percent of asymptomatic blood donors tested positive for Zika viral RNA [51]. The exact duration of viremia is unknown but is believed to last 1–2 weeks. Although early data on definitive proof of transfusion transmission were lacking, there have been credible cases reported from Brazil and Indochina [52,53]. Later, in a meta-analysis, the prevalence of Zika infection in blood donations was found to be at a high-risk level of 1.02%, with significant variations across different regions [51].

Preventive measures against the transfusion-transmitted Zika virus include temporarily deferring blood donors in epidemic areas, encouraging donors to report Zika virus symptoms after donation with or without quarantine of blood components, supplying blood collected from non-endemic regions to epidemic regions, implementing nucleic acid testing of blood donations, and employing pathogen inactivation methods for blood products. In endemic areas, all blood donors are at risk of infection, making it impractical to import blood from low-risk regions to supply most endemic areas due to logistical challenges and the large volume of blood required. Donor deferral and post-donation reporting are limited by the high rate of asymptomatic infections. Pathogen inactivation methods capable of adequately reducing flavivirus levels are only available for plasma and platelets, while red blood cells constitute the majority of transfused blood components [54].

NAT blood screening tests and post-donation surveillance are recommended in Zika virus-endemic regions, and appropriate strategies should be implemented based on specific conditions. Further research is needed to conduct more detailed studies in the future. The combination of detecting Zika virus RNA through polymerase chain reaction and post-donation surveillance may help reduce the risk of transmission through blood transfusions.

### 4.4. Dengue and Chikunguya Viruses

These viruses are primarily endemic in Africa and Asia; however, they are becoming an increasing concern in the Caribbean and the United States [55]. Similar to malaria and Zika viruses, they are transmitted through the bite of infected *Aedes* species mosquitoes. While the dengue virus and the chikungunya virus are not prevalent in Europe or the continental United States, outbreaks in Asia, Pacific countries, South and Central America, and Caribbean islands have raised concerns about transfusion transmission in those regions and among returning travellers [56,57].

Dengue and chikungunya fevers are generally considered less severe than other mosquito-transmitted diseases, as fatalities are rare and mainly occur among the elderly and infants. The approximate rate of transfusion transmission from dengue virus RNA-positive transfusions is 33% [58]. However, the incidence of dengue-virus-related symptoms in hospitalized patients infected through transfusion or other routes was similar to that in non-infected control patients, leading to the discontinuation of prospective NAT screening in several countries.

### 4.5. Ebola

To date, there have been no reported cases of Ebola virus transmission through donated blood, tissues, or organs [59]. However, asymptomatic infections with replicating Ebola virus have been documented [60,61]. As a precautionary measure, individuals who have traveled from Ebola-virus-affected countries are deferred from donating blood, as there is an overlap between countries at risk for malaria and those currently at risk for Ebola virus disease in Africa. Nevertheless, specific guidelines are necessary to ensure the safety of blood donation by individuals exposed to Ebola virus, particularly considering the potential for an Ebola virus disease outbreak to occur in regions without malaria risk.

### 4.6. West Nile Virus

West Nile virus (WNV), a flavivirus transmitted by mosquitoes, was first detected in the United States in 1999, initially infecting birds and horses and later causing human cases. Initially, it was believed that WNV transmission to humans occurred solely through infected mosquitoes. However, in 2002, it was discovered that transfusion transmission had resulted in WNV infection in more than 20 individuals [62]. Considering the widespread outbreaks that have occurred in the past two decades, WNV can no longer be regarded as a minor risk to human health but rather a global threat. Since 80 percent of WNV-infected individuals do not show symptoms, the only way to identify infected blood donors is by screening a blood sample using nucleic acid amplification technology-based tests [63].

Following the introduction of a nucleic-acid-based assay in the United States, the Centers for Disease Control and Prevention (CDC) conducted a screening program for WNV infection among blood donors [64]. Among approximately 1 million donations screened, a total of 163 donations repeatedly tested positive for West Nile virus, and these units were excluded from the blood supply. These findings indicate that investigational screening tests are successful in identifying donations containing the virus and preventing the introduction of implicated blood components into the blood supply [65]. In the absence of a NAT test, a 28-day deferral period may be an acceptable method.

### 4.7. Torque teno Virus

Torque teno virus (TTV) was initially identified in 1997 among Japanese patients who acquired hepatitis through blood transfusion [66]. TTV DNA has been detected in human plasma exosomes, leading to speculation that TTV may have the ability to spread intercellularly via these exosomes and be transmitted from one individual to another through blood transfusion [67]. Consistent with this finding, an observational study reported a high prevalence of TTV among healthy blood donors in the Brazilian population [68]. High incidence does not necessarily equate to the presence of infectivity. This caution is supported by the observation that recurrent blood transfusions did not increase TTV prevalence in beta thalassemia patients [69].

### 4.8. Sen Virus

A surprisingly large proportion of healthy donors are SEN-V (Sen virus) positive (approximately 10%) [70]. Ribavirin-based anti-hepatitis C virus therapies may be less effective with SEN-V positivity. The clinical importance of SEN-V virus transmission needs further exploration.

### 4.9. Parvovirus B19

Human parvovirus B19, also referred to as erythrovirus B19, is a small, non-enveloped single-stranded DNA virus [71]. It has a high resistance to commonly used inactivation methods, such as heat and solvent/detergent treatment [72]. Additionally, its small size makes it challenging to remove through filtration. Parvovirus B19 can be transmitted through blood components and certain plasma derivatives, posing a risk of morbidity to susceptible recipients, including pregnant women, individuals with hematological malignancies or hemolytic diseases, and immunocompromised individuals [73]. Approximately 30% of potential blood donors lack antibodies against parvovirus B19, rendering them susceptible to new infections. Since infections often go unnoticed, parvovirus B19-infected donors cannot be identified based on clinical symptoms or abnormalities in the blood count, allowing them to donate blood [74]. Consequently, the detection of parvovirus B19 DNA in blood donations is not uncommon. Following acute infection, parvovirus B19 viremia can persist for months to years in healthy blood donors. However, DNA concentrations decline rapidly after the acute phase and peak viremia, accompanied by the production of potentially neutralizing IgG antibodies. The most effective approach to prevent transfusion-transmitted parvovirus B19 infections is to implement routine screening of blood donors for parvovirus B19 DNA using NAT.

### 4.10. Cytomegalovirus

Transfusion-transmitted cytomegalovirus (CMV) is often asymptomatic; however, certain patient populations, such as pregnant women, patients with primary immunodeficiencies, transplant recipients, and patients undergoing chemotherapy or transplantation for malignant disease, may be at risk of life-threatening CMV infection [75,76].

Acute CMV infection in these high-risk populations can have severe consequences, highlighting the need for measures to reduce the risk of transfusion-transmitted CMV infection. Implementing universal leukoreduction significantly reduces the residual risk of CMV transmission through blood products [77,78].

Studies examining CMV infections in donors have revealed that newly seropositive donors have a higher prevalence and concentration of CMV DNA in both leukocytes and plasma compared to window-phase donations or donations from long-term seropositive donors. Furthermore, antibodies in the early seropositive stage of primary CMV infections do not exhibit neutralizing effects in vitro. Therefore, blood from donors during seroconversion may pose the highest risk of transfusion-transmitted CMV. This understanding can aid in designing additional strategies to further mitigate the risk of transmission in conjunction with leukoreduction. However, after a comprehensive review, the AABB did not issue clinical practice guidelines regarding the appropriate usage of leukoreduced and/or CMV seronegative blood products [79].

Transfusion of unselected blood components can lead to intermittent CMV IgG levels in previously seronegative patients due to the transfer of CMV IgG from seropositive leucoreduced blood products. This should be considered as a potential cause for the detection of new CMV IgG levels in transfused patients. Importantly, acquired CMV IgG levels should not lead to the misinterpretation of a CMV-positive hematopoietic stem cell transplant in an actually CMV-naïve recipient [80].

### 4.11. Ebstein–Barr Virus

Ebstein–Barr Virus (EBV), also known as human herpesvirus 4 (HHV-4), is a linear, double-stranded DNA virus, which infects up to 95% of the population by 40 years of age [81]. EBV persists lifelong in B-lymphocytes, with clinically relevant risk of reactivation in immunocompromised individuals. EBV spreads most commonly through bodily fluids, especially saliva. However, EBV can also spread through blood and semen during sexual contact, blood transfusions, and organ transplantations. EBV genomes are detectable in viable B-cells for the duration of RBC storage [82]. Although studies have identified viral nucleic acids in donor blood, the risk of transfusion transmission is controversial. There is limited evidence demonstrating its ability to cause transfusion-transmitted infection in recipients, particularly since many individuals already possess latent infections that are effectively controlled by their immune systems [83]. The B-cell-associated nature of EBV and the implementation of universal leukocyte reduction methods significantly reduced the risk of transfusion transmission of the virus [84].

### 4.12. Human herpesvirus 8

Human herpesvirus 8 (HHV-8) is also a double-stranded DNA virus, sharing several similarities with EBV. While the seroprevalence is lower than that of EBV (1–5% in non-endemic regions), its clinical significance stems from the fact that HHV-8 is etiologically associated with various types of Kaposi’s sarcoma (KS) and other uncommon neoplastic conditions such as primary effusion lymphoma (PEL), solid organ variants of KS, and Castleman disease (MCD) [85].

HHV-8 can be found in peripheral blood mononuclear cells. However, the presence of free HHV-8 in plasma is extremely rare, except in immunosuppressed HIV-infected patients [82,86]. With the use of universal leukocyte reduction methods, the risk of transfusion transmission is negligible.

### 4.13. Human T-lymphotropic Virus I and II

Human T-lymphotropic virus (HTLV) types I and II are retroviral infections that primarily target T-cells. These viruses exhibit a strong association with lymphocytes, leading to persistent infections that often remain asymptomatic. Although most HTLV-infected individuals do not develop any symptoms, around 2% to 5% of those infected with HTLV-I may experience the onset of adult T-cell leukemia/lymphoma after a latency period of 20 to 30 years [87].

In the United States, the seroprevalence rates of HTLV-I/II among volunteer blood donors average around 0.016% [88]. Transmission of HTLV-I through blood transfusion is primarily associated with the transfusion of cellular blood components, including whole blood, red blood cells, and platelets, while transmission via plasma fractions or derivatives from HTLV-I-infected blood is rare [89]. The likelihood of transmission through whole blood or packed red blood cells appears to decrease with longer storage durations and the implementation of leukoreduction techniques.

### 4.14. SARS-CoV-2

Shallow transmission risk might be due to no binding site for COVID-19. Some anecdotic data are available of practically no transmission with convalescent donors [90]. The more significant problem was fewer donations with the pandemic events. However, precautionary measures are still recommended, such as deferral from blood donation for 21 days after any possible exposure to patients with confirmed infection, and those recovering from COVID-19 should avoid donating blood for at least 28 days after the resolution of their symptoms. Also, the importance of plasma collection from convalescent donors must be emphasized, since convalescent plasma is a safe and effective treatment for COVID-19 in immunocompromised patients [91].

### 4.15. Monkeypox Virus

While transfusion transmission of monkeypox virus (MPXV) has not been reported, the potential of transfusion transmission seems to be supported by evidence [92,93,94]. However, it is currently not recommended for blood banks to ask donors specific questions about possible exposure to the monkeypox virus or to use diagnostic tests to screen patients and blood products for monkeypox virus [95].

## 5. Areas of Particular Interest

### 5.1. Hemophilia

There was a long debate about whether gene synthetic coagulation factors (somehow slightly foreign proteins) induce more or fewer factor VIII inhibitors than plasma-derived concentrates. However, it was clear from the beginning that gene synthetic molecule concentrates are free of transfusion-transmitted pathogens. At the same time, plasma-derived factor concentrates were gained from many donors, carrying some risk of transmittable pathogens, even with high-quality screening and inactivation [1,3,4,96]. However, with the more and more prevalent use of procoagulant agents factor mimetics, like desmopressin, recombinant, activated factor VIIa, emicizumab, or fitusiran, the so-called aptamers, serine protease tissue factor modifiers are probably primarily free of human plasma protein or derivatives, so disease transmission in hemophiliacs (a significant problem some decades before) probably will no longer be a major issue, of course mostly in prophylactic therapeutic modalities. However, acute bleedings still need plasma-derived factor administration. The same is true for therapeutic gene efforts [2].

### 5.2. Bone Marrow Transplantation

Organ and bone marrow transplantation is a particular area of interest, as these patients are heavily immunocompromised, immunosuppressed, and at a high risk of typical or atypical, unusual infections [17,97,98]. Furthermore, an additional risk may appear in the case of bone marrow transplantation, with donor-derived or contaminated pathogen transmission originating from or during the stem cell harvesting or preparing process [99,100,101,102].

Transfusion need is generally large, but we should avoid more than necessary transfusions and provide platelet transfusions only for non-bleeding (non-febrile) patients with platelet counts of 10 G/L or less or under 20 g/L in patients with fever according to international guidelines [103]. The new data of cooled platelet transfusion options (see previously) with less infection transmission risk might need new considerations whether we encounter a bleeding episode or recommend a prophylactic measure. The same is true for the hemoglobin 70 g/L threshold in most instances. In addition, try to reduce synchronous parenteral nutrition (if possible) in patients with excellent transfusion needs [17,65,98,104,105,106,107,108,109].

The infection risk is generally higher with horse or rabbit anti-human thymocyte immunoglobulins (ATG) [1,41,110,111]. Viral reactivation in CMV, BK, and EBV is somewhat higher in ATG-conditioned or treated patients. Probably, there is a slight increase in post-transplant lymphoproliferative disease as well. However, the production process of these products ensures proper bacterial and viral inactivation steps, so disease transmission risk is minimal with these products.

The administration of allogenic grafts to the recipients confers the risk of infectious and non-infectious disease transmission [48,98,99,108]. This risk can be minimized by thorough donor testing. The donor testing for the presence of infectious diseases should be done by serology and combined NATs. Also, the window period from the donor testing to the actual cell procurement ensures safety as diseases tested negative initially may become detectable during this window period. Therefore, a minimum of two weeks is advisable for stem cell donors [37,96,98,99,107,112].

Donors are routinely tested for hepatitis A, B, and C viruses, HIV, EBV, CMV, Toxoplasma, and Treponema infections, minimizing the risk of these transmissions [37,78,80,99,102].

Cytomegalovirus has a unique aspect as the population carries this virus in their lymphatic cells, and thus very often, the transmission cannot be avoided in transplant settings. However, proper screening and prophylaxis with new drugs, such as letermovir, can reduce the risk of severe reactivation and infection in these patients [65].

Some transmission hazards need even more careful attention in bone marrow transplant patients. West Nile fever is a single strain RNA flavivirus of particular interest, as the disease can be transmitted with blood products. The disease is usually asymptomatic in 75% of cases. Thus, the donor is not aware of any deferring condition. NAT testing for the presence of this virus is mandatory in areas where the occurrence is expected or if the donors visited these areas three months before donation. The importance is that the severe neuroinvasive form of this disease is present in 1% of all cases but is more common in immunosuppressed (transplant) patients [18,65,102,112].

Endemic areas for special arboviruses should test for the presence of chikungunya, Zika, and dengue viruses, causing fewer problems. Also, donors who visited these areas before donation should be tested or temporarily deferred until incubation time has elapsed. Zika-virus-positive donors should be deferred for a minimum of 120 days from stem cell donation, as the viral clearance is slow even in asymptomatic patients. A retest is compulsory with sensitive NAT to confirm donor availability. Chikungunya virus infection is a flu-like self-limited disease. Therefore, donors should be deferred for at least 30 days before procurement. Dengue virus tests are cross-reacting with Zika tests, so a specific NAT test is always preferred, and positive donors should be deferred for a minimum of 60 days or until the NAT test is negative [102,113,114,115].

Transplantation-associated monkeypox transmission [92], malaria-falciparum-induced hemophagocytic syndrome [23], Cache Valley virus transmission in kidney transplant patients, severe fever thrombocytopenia syndrome [19], and non-BK reactivation-induced hemorrhagic cystitis [116], even if rare, are also essential observations in these cohorts of patients.

Furthermore, a few recently published cases have described potential blood-borne transmission risks that often receive less attention outside of transplant and heavily immunosuppressed settings [117]. These cases highlight the need for new efforts to explore donor selection from different perspectives. The conditions of concern include rabies, brucellosis, melioidosis (Whitmore disease), and leptospirosis.

Rabies is well known and easily identifiable based on clinical signs and medical history. Brucellosis, initially associated with cows and dairy products, can also spread to dogs with mild clinical signs. Dog owners can become infected, and transmission through cell therapy raises concerns about inducing disease in recipients, necessitating further clarification.

Interestingly, leptospirosis can be associated with uncommon donor behaviors, such as participation in adventurous recreational activities like rafting and rock-climbing. If transmitted, this disease can provoke severe illness in the recipient. On the other hand, melioidosis poses a lesser threat but still carries transmission potential. It is not limited to tropical areas, as cases have been reported in the southern part of the United States.

Pathogen reduction efforts and blood product irradiation are essential in reducing transfusion-induced graft versus host disease in general and in transplant settings [118].

### 5.3. Changes in Screening and Inactivation Methods

The importance of donor selection and interview cannot be emphasized enough [1,3,5,9,20,112]. A careful look at the patient’s lifestyle and travel, anamnesis, sexual attitudes, social backgrounds, and previous or present disorders remain standard elements of donor selection to identify safe donors who should wait before donation and be quarantined for a while. The inventory of traditional microbiological screening methods and high-tech analysis like NAT, PCR, and genetics should always be performed and refreshed in due time.

New-generation inactivation methods became standard elements of European preparative transfusiology standards, in which chemical intervention combined with ultraviolet light became a widely applied method in many European countries [1,4,22,41,42,94,96,102].

The increasing prevalence of diverse pathogens and their emergence in unforeseen circumstances, such as travel and climate changes, necessitates ongoing endeavors to enhance pathogen inactivation methods. These efforts primarily focus on impeding the replication of various bacteria, viruses, and other pathogens through the utilization of conventional techniques as well as newer approaches, such as INTERCEPT, MIRASOL, THERAFLEX-platelets, and S-303, among others [119]. These methods establish a robust and vital foundation for fostering collaboration between blood banking personnel and clinicians within the realm of clinical transfusiology.

### 5.4. Climate Change

The dynamics of pathogen transmission in transfusion medicine have been notably altered by climate change, with mechanisms including population dynamics and extreme weather events, among others [120]. However, our understanding of these mechanisms is still incomplete. The emergence of previously unexpected infections necessitates the expansion and refinement of donor interviews and laboratory screening processes. These steps are crucial due to the potential for these infections to be asymptomatic or present atypical symptoms, such as in the case of the hepatitis E virus and Zika. It is important to note that the pathogen inactivation methods employed in stem cell preparation procedures may differ from those used in blood banking, highlighting the need for careful consideration and adaptation in these contexts.

## 6. Conclusions

The continuous evolution of data and changes in the geographical distribution of transfusion-transmissible pathogens require coordinated efforts in the field of clinical transfusiology. It is essential for clinicians to regularly update and gather important and relevant information related to this topic. The evolving challenges in donor selection for different bone marrow transplant strategies, constrained by limited time frames and shifting geographical patterns of well-known pathogens, as well as the emergence of unexpected pathogens or disease entities that may be transmitted through stem cell infusions, necessitate ongoing vigilance and regularly updated knowledge and skills in these areas.

This review aims to draw attention to the dynamic nature of this scenario and promote an individualized medicine approach in the field. Promising advancements can be seen in the application of individualized platelet support within transplant settings.

## Figures and Tables

**Table 1:** Symptoms and laboratory changes in acute hemolytic transfusion reaction, severe acute allergic reaction, and microbial contamination from transfusion.

	Acute Hemolytic Transfusion Reaction	Severe Acute Allergic Reaction	Microbial Contamination from Transfusion
**Symptoms**			
Fever	+	+	+
Chills	+	+	+
Dyspnoea	+	+	+
Tachycardia	+	+	+
Hypotension	+	+	+
Shock	+	+	+
Nausea, vomiting	+	+	+
Diarrhoea	+	+	+
**Laboratory Changes**			
Hemolysis	+	-	-
IgA-antibodies	-	+	-
Elevated inflammatory markers (CRP, PCT, IL-6)	-	-	+

IgA—immunoglobulin A, CRP—C-reactive protein, PCT—procalcitonin, IL-6—interleukin-6.
